# Total body irradiation versus chemotherapy before stem cell transplantation in children aged two to four with acute lymphoblastic leukemia—an EBMT pediatric diseases working party study

**DOI:** 10.1038/s41375-026-03114-x

**Published:** 2026-08-21

**Authors:** Anna Eichinger, Zofia Szmit, Katharina Kleinschmidt, Mouad Abouqateb, Marc Ansari, Peter Bader, Adriana Balduzzi, Friso Calkoen, Arnaud Dalissier, Jean-Hugues Dalle, Cristina Diaz-de-Heredia, Franca Fagioli, Maura Faraci, Jacques-Emmanuel Galimard, Brenda Gibson, Bernd Gruhn, Peter Lang, Oana Mirci-Danicar, Richard Mitchell, Christina Peters, Kanchan Rao, Samppa Ryhänen, Martin Sauer, Petr Sedlacek, Jan Styczynski, Selim Corbacioglu, Krzysztof Kalwak

**Affiliations:** 1 Department of Pediatrics, Dr. von Hauner Children’s Hospital, LMU University Hospital, LMU Medizin, Ludwig-Maximilians-Universität München, Munich, Germany; 2 Department of Pediatric Hematology, Oncology and BMT, Wroclaw Medical University, Wroclaw, Poland; 3 Department of Pediatric Oncology, Hematology and Stem Cell Transplantation, University Children’s Hospital Regensburg, Regensburg, Germany; 4 EBMT Paris Study Unit, Paris, France; 5 CANSEARCH Research platform for pediatric oncology and hematology, Faculty of Medicine, Department of Pediatrics, Gynecology and Obstetrics, University of Geneva, Geneva, Switzerland; 6 Division of Pediatric Oncology and Hematology, Department of Women, Child and Adolescent, University Geneva Hospitals, Geneva, Switzerland; 7 Division for Stem Cell Transplantation and Immunology, Department of Pediatrics, Goethe University, Frankfurt am Main, Germany; 8 Pediatrics, Fondazione IRCCS San Gerardo dei Tintori, Monza, Italy; 9 University of Milano-Bicocca, School of Medicine and Surgery, Monza, Italy; 10 Princess Maxima Center, Utrecht, The Netherlands; 11 Hôpital Robert Debre, Pediatric Hematology and Immunology Department, GHU AP-HP Nord – Université Paris Cité, Paris, France; 12 Department of Pediatric Hematology and Oncology, Hospital Universitari Vall d’Hebron, Barcelona, Spain; 13 Pediatric Onco-Hematology Department and Stem Cell Transplantation and Cellular Therapy Division, Regina Margherita Children’s Hospital, University of Turin, Turin, Italy; 14 Department of Public Health and Pediatrics, University of Turin, Turin, Italy; 15 HSCT Unit, IRCCS Istituto Giannina Gaslini, Genova, Italy; 16 Department of Paediatric Haematology, Royal Hospital for Sick Children, Glasgow, UK; 17 Department of Pediatrics, Jena University Hospital, Jena, Germany; 18 Department of Hematology and Oncology, University Children’s Hospital, Eberhard Karls University Tuebingen, Tuebingen, Germany; 19 Bristol Royal Hospital for Children Dept. of Paediatric Haematology/Oncology/BMT, Bristol, UK; 20 Royal Manchester Children’s Hospital, Paediatric BMT, Manchester, UK; 21 Kids Cancer Centre, Sydney Children’s Hospital, Randwick, NSW Australia; 22 School of Women’s & Children’s Health, UNSW, Sydney, NSW Australia; 23 St. Anna Children’s Hospital, University Vienna, St. Anna Children’s Cancer Research Institute, Vienna, Austria; 24 Great Ormond Street Hospital for Children NHS Foundation Trust, London, UK; 25 Children’s Hospital, University of Helsinki and Helsinki University Hospital, Helsinki, Finland; 26 Pediatric Hematology and Oncology, Hannover Medical University, Hannover, Germany; 27 University Hospital Motol, Department of Paediatric Haematology and Oncology, Prague, Czech Republic; 28 Department of Pediatric Hematology, Oncology, Immunology and Transplantology; Collegium Medicum, Nicolaus Copernicus University, Bydgoszcz, Poland; 29 Present Address: Department of Pediatrics, Medical University Lausitz – Carl Thiem, Cottbus, Germany

**Keywords:** Paediatrics, Leukaemia, Medical research

## To the editor

Total body irradiation (TBI)-based conditioning is the standard preparative regimen before allogeneic hematopoietic stem cell transplantation (alloHSCT) in children with acute lymphoblastic leukemia (ALL) older than 4 years, whereas chemotherapy (CTx)-based approaches are generally preferred in children younger than 2 years because of concerns regarding long-term toxicity [1]. For children aged 2 to 4 years, however, the optimal conditioning strategy remains undefined. This age group is clinically important, as ALL incidence peaks in early childhood, and treatment decisions must balance relapse prevention and survival against the risk of long-term side effects, including subsequent malignancies (SMNs) during a vulnerable developmental period.

We therefore performed a retrospective multicenter analysis within the EBMT Pediatric Diseases Working Party to evaluate outcomes after first alloHSCT in children with ALL aged 2 to 4 years. Patients underwent HSCT in first or second complete remission (CR1/2) between 2000 and 2012 after myeloablative TBI-based or CTx-based conditioning (supplementary fig. 1). The primary endpoint was SMN incidence. Key secondary endpoints were overall survival (OS), leukemia-free survival (LFS), relapse incidence (RI), non-relapse mortality (NRM), graft-versus-host disease (GVHD), and refined GVHD -free, relapse-free, and event-free survival (GRFS), defined as survival without grade III-IV aGVHD, extensive cGVHD, relapse, SMN, or post-transplant lymphoproliferative disorder (PTLD), thereby incorporating SMN events into the overall assessment of conditioning strategies.

A total of 280 patients met study criteria, of whom 161 (57.5%) received TBI-based and 119 (42.5%) CTx-based conditioning. Baseline characteristics were largely comparable between groups. Disease status at transplantation, leukemia risk factors, and donor type did not differ significantly between groups. However, graft source differed, with bone marrow used more frequently in the TBI group (72.0% vs. 52.9%) and peripheral blood stem cells (PBSC) more often in the CTx group (26.1% vs. 9.3%, *p* < 0.001). Median follow-up was 13.2 years and did not differ significantly between the two conditioning groups, permitting comparison of late events. TBI was combined with different chemotherapeutic agents, most frequently cyclophosphamide (49.0%) or etoposide (42.9%); radiation doses ranged from 7 to 16 Gy, with 70.2% receiving 10.0 to 12.0 Gy. A variety of myeloablative CTx-based regimens were used, most commonly busulfan-based (86.4%) and treosulfan-based (10.1%). Detailed patient, disease, transplant, conditioning, and GVHD prophylaxis characteristics are given in Supplementary Table 1.

**Fig. 1:**
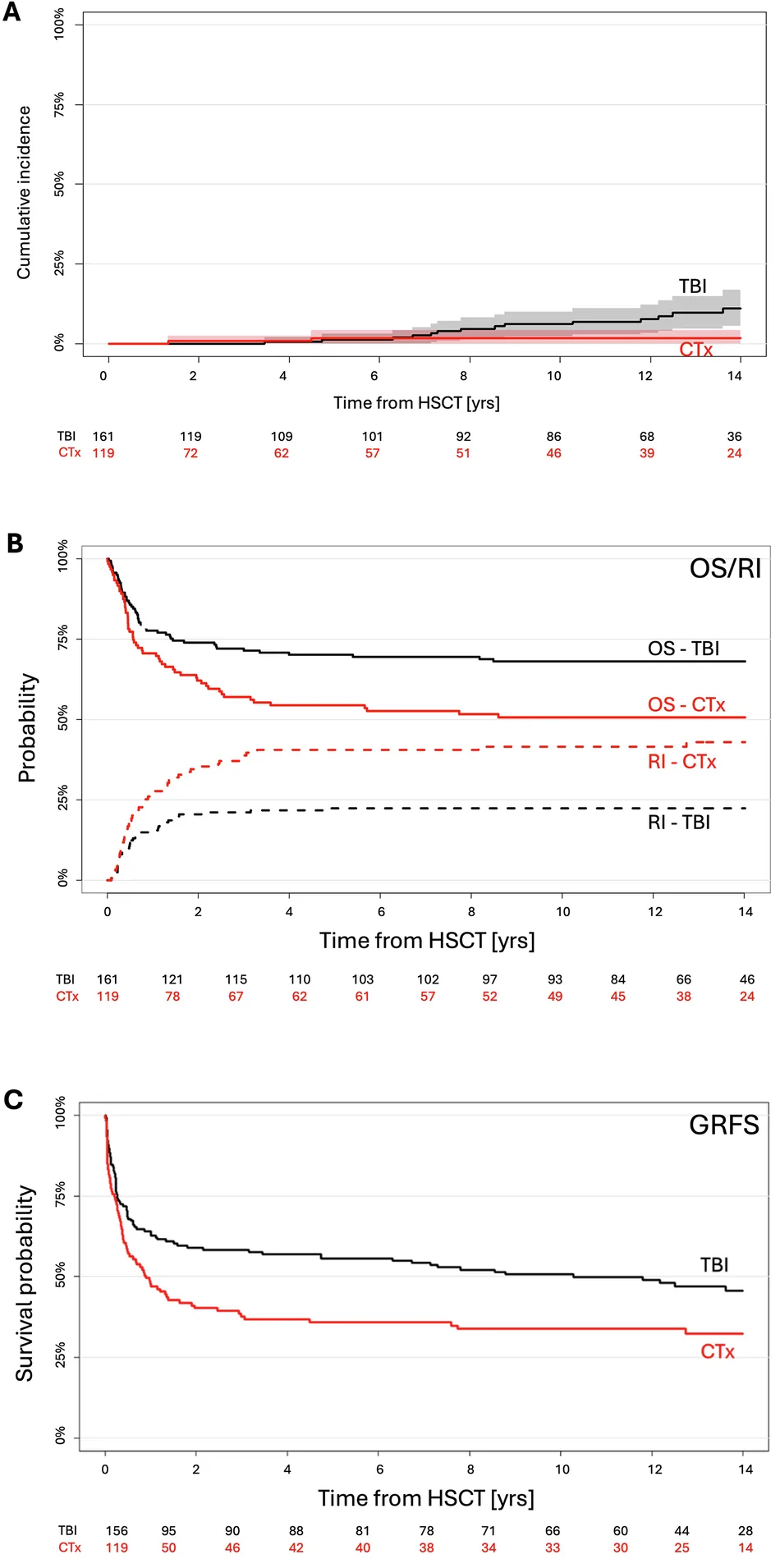
HSCT outcomes. CI of SMN (excluding PTLD; **A**), OS probability and RI (**B**) and refined GRFS (**C**) over time [years after HSCT]. Subgroups are indicated via color code: CTx red, TBI black. In **B**, numbers at risk correspond only to OS analysis. HSCT hematopoietic stem cell transplantation, CTx chemotherapy, GRFS graft-versus-host-disease-free, relapse-free, event (including SMN and PTLD)-free survival, OS overall survival, PTLD post-transplant lymphoproliferative disease, RI relapse incidence, SMN subsequent malignant neoplasm, TBI total body irradiation.

Seventeen solid SMNs and two cases of PTLD were reported in the overall cohort (6.8% of all patients). No cases of SCC were observed in our cohort, likely due to the young age, relatively low cGVHD incidence, and limited event numbers [2]. No secondary myeloid malignancies were reported, despite etoposide use in nearly 90% of conditioning regimens [3]. The 13-year cumulative incidence of SMNs was 6.4% overall and thus in line with previously published reports, but differed substantially by conditioning regimen, reaching 9.7% after TBI versus 1.8% after CTx [4, 5] (Fig. 1A). PTLD occurred only in the CTx group and at short latency (0.5 and 2 years). By contrast, median latency to SMN or PTLD was substantially longer after TBI than after CTx (8.5 years [Q1;Q3][6.9;12.0] versus 1.6 years [Q1;Q3][1.1;2.6]; Mann-Whitney *p* < 0.001), consistent with the delayed emergence expected for radiation-associated solid tumors.

Additional information on whether diagnosis was triggered by symptoms or screening was available for only six patients. Four SMNs, all thyroid carcinomas, were diagnosed by routine annual ultrasound screening, whereas two were diagnosed after symptom-triggered investigations (*n* = 1 osteosarcoma: visible mass, *n* = 1 glioblastoma: headache). Among patients with SMN or PTLD, none with available treatment data had received a CNS boost (SMN, *n* = 10/17, including both patients with glioblastoma). Thyroid carcinoma, for which TBI is a well-established risk factor, accounted for approximately half of all SMNs and was—as expected—generally associated with favorable outcomes in our cohort [6]. In contrast, aggressive SMNs occurred as well, including glioblastoma and osteosarcoma. By the end of follow-up, three patients with SMN/PTLD had died from glioblastoma (*n* = 2) or PTLD (*n* = 1). Thus, while the overall SMN incidence was within the expected range, these events were associated with substantial morbidity and mortality. Additional information regarding SMNs/PTLDs can be found in Table 1 and supplementary table 2, 3.

**Table 1:** Type of SMN/PTLD according to treatment group (CTx, TBI) and current patient status (dead/alive).

Type of SMN/PTLD	CTx	TBI	Total	Treatment	Alive at last FU
Secondary myeloid neoplasm	0 (0.0%)	0 (0.0%)	0 (0.0%)	–	–
Thyroid carcinoma	1 (25.0%)	9 (60.0%)	10 (52.6%)	Thyroidectomy (*n* = 2); surgery + radioactive iodine therapy (*n* = 3); surgery including neck dissection + iodine therapy (*n* = 1); n/a (*n* = 4)	100%
SCC	0 (0.0%)	0 (0.0%)	0 (0.0%)	–	–
Glioblastoma	0 (0.0%)	2 (13.3%)	2 (10.5%)	Surgery + radiotherapy + CTx (*n* = 1); surgical biopsy + CTx (*n* = 1)	0%
Intestinal polyposis^a^	0 (0.0%)	1 (6.7%)	1 (5.3%)	Polypectomy only (*n* = 1)	100%
Melanoma	0 (0.0%)	1 (6.7%)	1 (5.3%)	n/a (*n* = 1)	100%
Neurinoma	0 (0.0%)	1 (6.7%)	1 (5.3%)	Surgery only (*n* = 1)	100%
Osteosarcoma	1 (25.0%)	1 (6.7%)	2 (10.5%)	Surgery + CTx (*n* = 1), n/a (*n* = 1)	100%
PTLD	2 (50.0%)	0 (0.0%)	2 (10.5%)	Rituximab (*n* = 1), n/a (*n* = 1)	50%
Total	4	15	19	12 (n/a *n* = 7)	16/19 alive

Additional information on SMNs/PTLDs can be found in supplementary table 1. ^a^with signs of malignant transformation.

*CTx* chemotherapy, *n/a* no information available, *NHL* Non-Hodgkin lymphoma, *PTLD* post-transplant lymphoproliferative disorder, *SCC* squamous cell carcinoma, *SMN* subsequent malignant neoplasm, *TBI* total body irradiation.

TBI-based conditioning was associated with superior long-term leukemia control and survival. Ten-year OS was 68.1% after TBI versus 50.7% after CTx, and 10-year LFS was 64.4% versus 44.0%, respectively (Fig. 1B). Ten-year-RI was markedly lower after TBI (22.4% vs 41.5%), whereas NRM was similar between groups (13.2% vs 14.4%). In multivariable analysis, CTx-based conditioning remained significantly associated with inferior OS (HR 1.77, 95% CI 1.19–2.63; *p* = 0.005), inferior LFS (HR 1.92, 95% CI 1.33–2.77; *p* < 0.001), and higher RI (HR 2.31, 95% CI 1.48–3.61; *p* < 0.001; supplementary table 4 and supplementary fig. 2). These findings suggest that the principal advantage of TBI in this cohort was improved leukemia control without an apparent increase in transplant-related mortality, consistent with observations in cohorts of older patients [1].

This pattern was also reflected in refined GRFS (Fig. 1C). Thirteen-year GRFS was 47.1% after TBI and 32.5% after CTx, and CTx-based conditioning was associated with significantly worse GRFS in multivariable analysis (HR 1.54, 95% CI 1.11–2.13; *p* = 0.009; supplementary table 6). Cell recovery as well as acute and chronic GVHD rates did not differ significantly between groups (supplementary table 5 and supplementary fig. 3). Taken together, these data suggest that the benefit of TBI in children aged 2 to 4 years extends beyond OS alone and is also reflected in improved refined GRFS. However, the improved survival observed with TBI must be interpreted not only in the light of a higher burden of SMNs, but also in the context of previous reports showing reduced life expectancy in pediatric cancer survivors because of increased risks of cardiovascular complications, cerebrovascular disease, and frailty [7]. In addition, SMN incidence continues to increase with age, including the occurrence of “adult-type” malignancies such as colon carcinoma [8]. These findings highlight the importance of educating families and survivors about long-term risks. Lifestyle interventions, vaccination strategies (e.g., HPV, hepatitis B), and comprehensive therapy summaries should be part of long-term follow-up [9, 10].

Long-term neurological toxicities, which may be more pronounced after TBI, were not assessed in this study. Unfortunately, collection of this data was not feasible within the registry-based design of our study. The severity of these effects and long-term impact on independent living remain uncertain, with mixed results in previous studies [11]. These aspects are important considerations, particularly in patients with pre-existing neurological impairments. A study by Neven et al. complements these findings by comparing long-term physical, educational, and quality-of-life outcomes in former ALL patients from the LEA long-term follow-up program [12]. That study does not show greater long-term toxicity after TBI-conditioning for alloHSCT in children aged 2 to 4 years in comparison with those transplanted at ≥4 years of age. Together, these data may help to further refine the debated benefit-risk balance of TBI in this age group.

Study limitations include the retrospective design, partial EBMT center participation, and potential selection bias. Differences in graft source, age, and pre-existing morbidities between groups may have influenced conditioning choice and outcome. Data on family history, germline mutations, cumulative therapy exposure, and details of TBI administration were not uniformly available. These limitations are particularly relevant for the interpretation of SMN risk, which is likely multifactorial, shaped by an interplay of cumulative treatment and host susceptibility. TBI dose and chemotherapeutic agents used for conditioning also varied within the cohort, and dose-response or chemotherapy-subtype analyses for SMNs were not feasible because of the limited number of events. Data on measurable residual disease (MRD) were not available for most patients in this cohort and could therefore not be accounted for in LFS/RI analyses.

Optimizing upfront ALL treatment protocols to minimize the need for consolidation with alloHSCT remains crucial to balance long-term toxicity with leukemia control. MRD is now a well-established marker guiding the decision between HSCT and chemotherapy alone in relapse treatment protocols for ALL. Moreover, different immunotherapeutic approaches have been successfully incorporated into upfront and relapse protocols and are reshaping the role of HSCT in ALL [13]. Research aimed at optimizing CTx-conditioning regimens is also ongoing. A small matched sub-study within the FORUM trial suggested that younger patients (<4 years of age) may benefit from pharmacokinetically optimized busulfan dosing (AUC 73.3-98.0 mg*h/l), achieving comparable OS and EFS with lower RI [14]. In addition, a recent retrospective study, in which more than half of the patients underwent pharmacogenetic monitoring, also reported comparable EFS and OS for the TBI and CTx groups [15].

At present, TBI-based conditioning remains an important option for very high-risk pediatric ALL patients aged 2 to 4 years, given its association with improved OS, lower RI, and improved refined GRFS. Treatment decisions should carefully balance these benefits against long-term toxicities, including SMNs and potential neurocognitive effects, and should consider individual risk factors such as genetic predisposition, neurodevelopmental status, and cumulative therapy exposure.

## Supplementary information

Supplementary Material

Supplementary Table 1

## Data Availability

Original data will be shared upon reasonable request; please contact Krzysztof.kalwak@gmail.com.
