# Supplementary material for: Total body irradiation versus chemotherapy before stem cell transplantation in children aged two to four with acute lymphoblastic leukemia—an EBMT pediatric diseases working party study
Source: Leukemia. 2026 Aug 21;40(11):2607–10. doi: 10.1038/s41375-026-03114-x (PMC13713552; doi:10.1038/s41375-026-03114-x)

Supplementary Material

Supplementary Tables

Supp. Table 1. Patient-, donor- and transplant-related characteristics. The following statistical tests are used as indicated: [1]Pearson's Chi-squared test, [2]Kruskal-Wallis rank sum test. Abbreviations: AraC cytarabine, ATG anti-thymocyte globulin, BM bone marrow, Bu busulfan, Clo clofarabine, CR1/2 first/second complete remission, CsA ciclosporine A, Cy cyclophosphamide, Eto etoposide, Flu fludarabine, GVHD graft-versus-host-disease; Mel melphalan, MMF mycophenolate mofetil, MTX methotrexate, ph-/+ Philadelphia chromosome negative/positive, PBSC peripheral blood stem cells, Tacro tacrolimus, TBI total body irradiation, TCD T cell depletion, Thio thiotepa, Treo treosulfan, (U)CB (unrelated) cord blood, yr(s) year(s).

	Yr (HSCT)	Conditioning regimen	Disease status	Donor type	Donor cells	GVHD prevention regimen	SMN/ PTLD		
							Type	Latency in [yrs][1]	FU since diagnosis in [yrs]
1	2002	TBI + Mel	CR1	MSD	BM	CsA + MTX	Thyroid cancer	13.6	1.5
2	2002	TBI + Cy	CR1	MSD	BM	CsA	Thyroid cancer	8.6	9.7
3	2003	TBI + Eto	CR1	MSD	BM	CsA + MTX	Thyroid cancer	11.8	8.8
4	2005	TBI + Eto	CR1	UD	BM	CsA + MTX	Thyroid cancer	16.7	0.7
5	2005	TBI + Eto	CR2	UD	BM	CsA + MTX	Thyroid cancer	6.3	10.9
6	2006	TBI + Cy	CR1	MSD	BM	CsA + MTX	Thyroid cancer	12.2	3.5
7	2008	TBI + CyThio	CR2	UD	BM	CsA + MTX	Thyroid cancer	7.3	7.2
8	2009	TBI + Eto	CR2	UD	BM	CsA + MTX	Thyroid cancer	4.7	9.4
9	2011	TBI + Eto	CR1	UCB	CB	CsA + MTX	Thyroid cancer	7.8	3.7
10	2011	TreoFluThio	CR1	MSD	BM	CsA + MTX	Thyroid cancer	4.5	6.5
11	2004	TBI + Cy	CR1	UD	BM	CsA + MTX	Glioblastoma	6.7	1.5[2]
12	2010	TBI + CyEto	CR1	UCB	CB	CsA	Glioblastoma	7.1	1.4[2]
13	2007	TBI + CyThio	CR2	UCB	CB	CsA	Intestinal polyposis	8.8	6.3
14	2000	TBI + AraC	CR1	UD	BM	CsA + MTX	Melanoma	10.3	5.0
15	2002	TBI + Eto	CR1	MSD	BM	CsA	Neurinoma	12.5	8.0
16	2009	TBI	CR2	UD	BM	CsA + MTX	Osteosarcoma	3.5	10.7
17	2012	BuCyEto	CR2	UD	BM	CsA + MTX	Osteosarcoma	1.3	9.9
18	2009	BuCyEto	CR1	UCB	CB	CsA	PTLD	2.0	0.1[2]
19	2010	BuCyMel	CR2	UD	BM	CsA	PTLD	0.5	7.0

***Supp. table 2. Additional information on SMN/PTLD cases**. Out of the patients suffering from SMN with additional available information (n=9) four had received some type of not further specified tumor predisposition screening with one patient harboring an NF1 mutation of unclear significance (thyroid carcinoma; no clinical neurofibromatosis type I features) and five had not received any type of screening. [1]Time between alloHSCT an initial diagnosis of SMN/PTLD in [years]. [2]FU until death due to SMN/PTLD. Abbreviations: AraC cytarabine, BM bone marrow, Bu busulfan, CB cord blood, CR1/2 first/second complete remission, CsA cyclosporine A, Cy cyclophosphamide, Eto etoposide, Flu fludarabine, FU follow-up, GVHD graft-versus-host disease, HSCT hematopoietic stem cell transplantation, Mel melphalan, MSD matched sibling donor, MTX methotrexate, NHL Non-Hodgkin lymphoma, PTLD post-transplant lymphoproliferative disorder, SMN subsequent malignant neoplasm, TBI total body irradiation, Thio thiotepa, Treo treosulfan, UD unrelated donor, yr(s) year(s).*

SMN CI in [%]	CTx (95%CI)	TBI (95%CI)	All (95%CI)
5 year-CI SMN + PTLD	3.4 (1.1-8.0)	1.3 (0.3-4.2)	2.2 (0.9-4.5)
10 year-CI SMN + PTLD	3.4 (1.1-8.0)	6.1 (3.0-10.8)	5.0 (2.8-8.1)
13 year-CI SMN + PTLD	3.4 (1.1-8.0)	9.7 (5.4-15.6)	7.0 (4.2-10.8)
5 year-CI SMN only	1.8 (0.3-5.7)	1.3 (0.3-4.2)	1.5 (0.5-3.5)
10 year-CI SMN only	1.8 (0.3-5.7)	6.1 (3.0-10.8)	4.3 (2.3-7.3)
13 year-CI SMN only	1.8 (0.3-5.7)	9.7 (5.4-15.6)	6.4 (3.7-10.0)

***Supp. table 3. Cumulative incidence of SMN and PTLD and SMN only at 5-year, 10-year and 13-year FU.** Abbreviations: CI cumulative incidence, CTx chemotherapy, FU follow-up, PTLD post-transplant lymphoproliferative disorder, SMN subsequent malignant neoplasm, TBI total body irradiation.*

	OS		NRM		RI		LFS	
**Characteristics**	**HR (95% CI)**	**p**	**HR (95% CI)**	**p**	**HR (95% CI)**	**p**	**HR (95% CI)**	**p**
**Conditioning strategy** (Ref: TBI)								
**TBI**	-		-		-		-	
**CTx**	1.77 (1.19-2.63)	**0.005****	1.28 (0.65-2.49)	0.48	2.31 (1.48-3.61)	**<0.001*****	1.92 (1.33-2.77)	**<0.001*****
**Age at transplant [yrs]**	1.14 (0.82-1.59)	0.43	1.12 (0.64-1.98)	0.68	1.41 (0.97-2.04)	0.073	1.31 (0.96-1.78)	0.090
**HSCT yr by 3 yrs**	0.76 (0.65-0.90)	**0.001****	0.70 (0.53-0.92)	**0.012***	0.92 (0.77-1.10)	0.36	0.84 (0.72-0.98)	**0.028***
**Disease status at HSCT** (Ref: CR1)	-		-		-		-	
CR2	1.98 (1.35-2.92)	**<0.001*****	1.44 (0.75-2.77)	0.27	1.83 (1.18-2.81)	**0.006****	1.70 (1.19-2.44)	**0.004****
**Donor type** (Ref: Related donor)	-	**-**	-	-	-	**-**	-	**-**
Unrelated donor	1.18 (0.75-1.85)	0.47	2.06 (0.86-4.96)	0.11	0.96 (0.60-1.56)	0.88	1.18 (0.78-1.79)	0.44
UCB	2.26 (1.30-3.92)	**0.004****	4.70 (1.75-12.6)	**0.002****	1.08 (0.56-2.09)	0.81	1.72 (1.02-2.90)	**0.044***

Supp. table 4. Multivariate analysis for OS, NRM, RI und LFS. *Multivariate analyses were conducted using Cox-cause specific hazard models. P values were marked as indicated: *p<0.05, **p<0.01, ***p<0.001. Abbreviations: CI confidence interval, CR1/2 first/second complete remission, CTx chemotherapy, HR hazard ratio, HSCT hematopoietic stem cell transplantation, LFS leukemia free survival, NRM non-relapse mortality, OS overall survival, ref reference, RI relapse incidence, TBI total body irradiation, UCB unrelated cord blood, yr(s) year(s).*

	cGVHD		aGVHD-II/IV		aGVHD-III/IV	
**Characteristics**	**HR (95% CI)**	**p**	**HR (95% CI)**	**p**	**HR (95% CI)**	**p**
**Conditioning strategy** (Ref: TBI)	-		-		-	
CTx	1.25 (0.62-2.52)	0.53	1.05 (0.65-1.67)	0.85	1.44 (0.76-2.71)	0.26
**Age at HSCT [yrs]**	0.80 (0.45-1.42)	0.45	1.01 (0.70-1.46)	0.95	1.38 (0.80-2.38)	0.25
**HSCT yr by 3 yrs**	0.56 (0.42-0.76)	**<0.001*****	0.95 (0.79-1.15)	0.63	0.92 (0.70-1.20)	0.53
**Disease status at HSCT** (Ref: CR1)	-		-		-	
CR2	2.00 (1.00-3.99)	**0.049***	0.82 (0.53-1.27)	0.38	1.25 (0.67-2.33)	0.49
**Donor type** (Ref: Related donor)	-		-		-	
Unrelated donor	1.31 (0.61-2.80)	0.49	1.55 (0.94-2.56)	0.086	1.15 (0.54-2.43)	0.72
UCB	1.59 (0.53-4.80)	0.41	1.38 (0.71-2.65)	0.34	1.86 (0.76-4.50)	0.17

Supp. table 5. Multivariate analysis for GVHD-related outcomes. *Multivariate analysis was conducted using Cox cause-specific hazard models. P values were marked as indicated: *p<0.05, ***p<0.001. Abbreviations: CI confidence interval, CR1/2 first/second complete remission, CTx chemotherapy, a/cGVHD acute/chronic graft-versus-host-disease, HR hazard ratio, HSCT hematopoietic stem cell transplantation, TBI total body irradiation, UCB unrelated cord blood, yr(s) year(s).*

GRFS	HR (95% CI)	p
**Conditioning Strategy (reference TBI)**		
CTx	1.54 (1.11-2.13)	**0.009****
**Age at HSCT**	1.18 (0.89-1.55)	0.24
**HSCT yr by 3 yrs**	0.83 (0.72-0.95)	**0.006****
**Disease Status at HSCT (reference CR1)**	-	
CR2	1.66 (1.21-2.29)	**0.002****
**Donor type (reference related donor)**	-	
Unrelated donor	1.08 (0.75-1.55)	0.69
UCB	1.56 (0.98-2.48)	0.059

Supp. table 6. Multivariate analysis for GRFS (as defined in methods). *Multivariate analysis was done using Cox. Analysis. P values were marked as indicated: *p<0.05, **p<0.01, ***p<0.001. Abbreviations: CI confidence interval, CR1/2 first/second complete remission, CTx chemotherapy, GRFS graft-versus-host-disease-, relapse-, event-free survival, HR hazard ratio, HSCT hematopoietic stem cell transplantation, TBI total body irradiation, UCB unrelated cord blood, yr(s) year(s).*

Supplementary Figures

Supp. figure 1.Flow diagram for inclusion and exclusion of patient population. *Abbreviations: ALL acute lymphoblastic leukemia, CR complete remission, HSCT hematopoietic stem cell transplantation.*

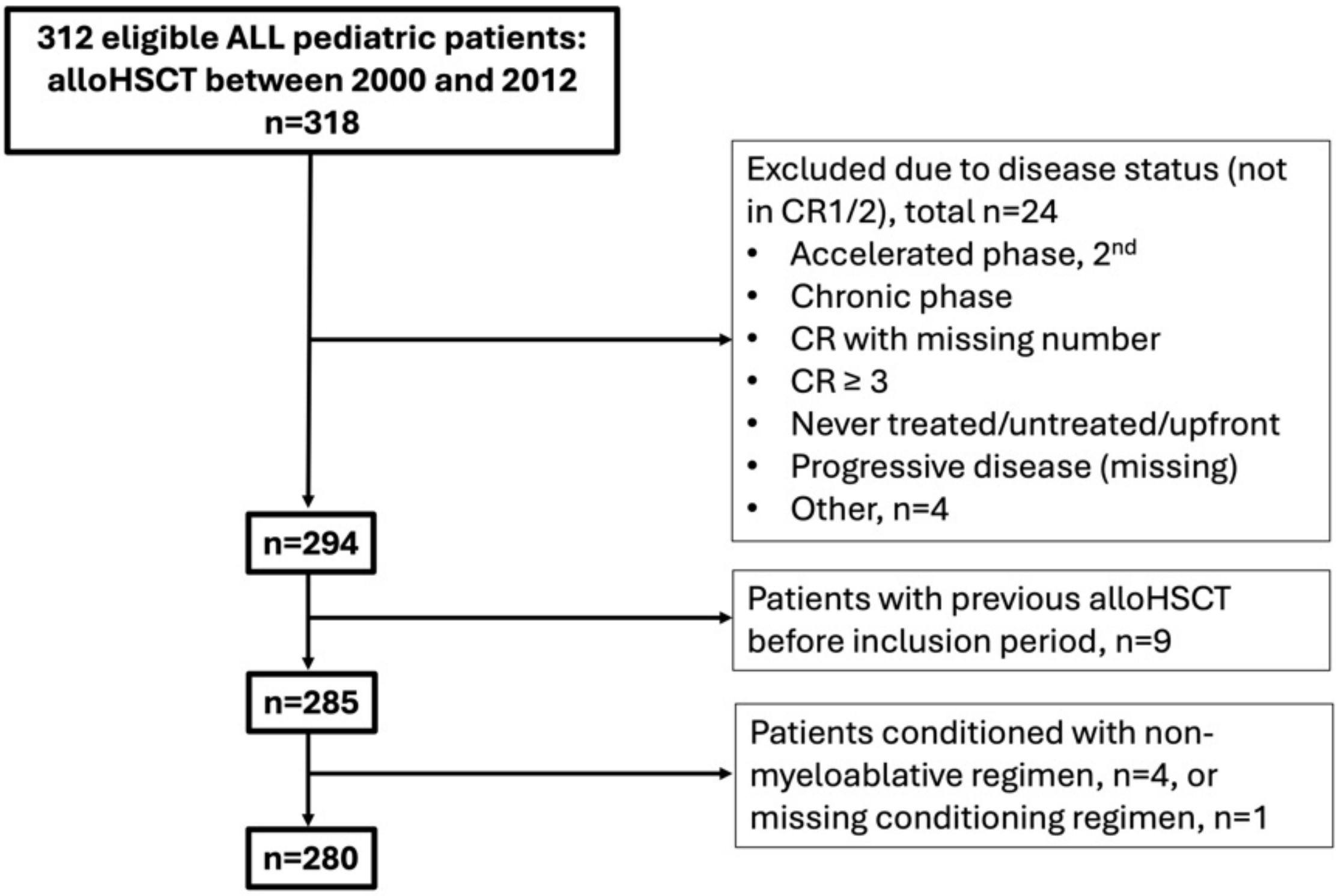

Supp. Figure 2. LFS (A) and NRM (B) following alloHSCT. *Subgroups are indicated via color-code: CTx red, TBI black. Abbreviations: CTx chemotherapy, HSCT hematopoietic stem cell transplantation, LFS leukemia free survival, NRM non-relapse mortality, TBI total body irradiation, yrs years.*

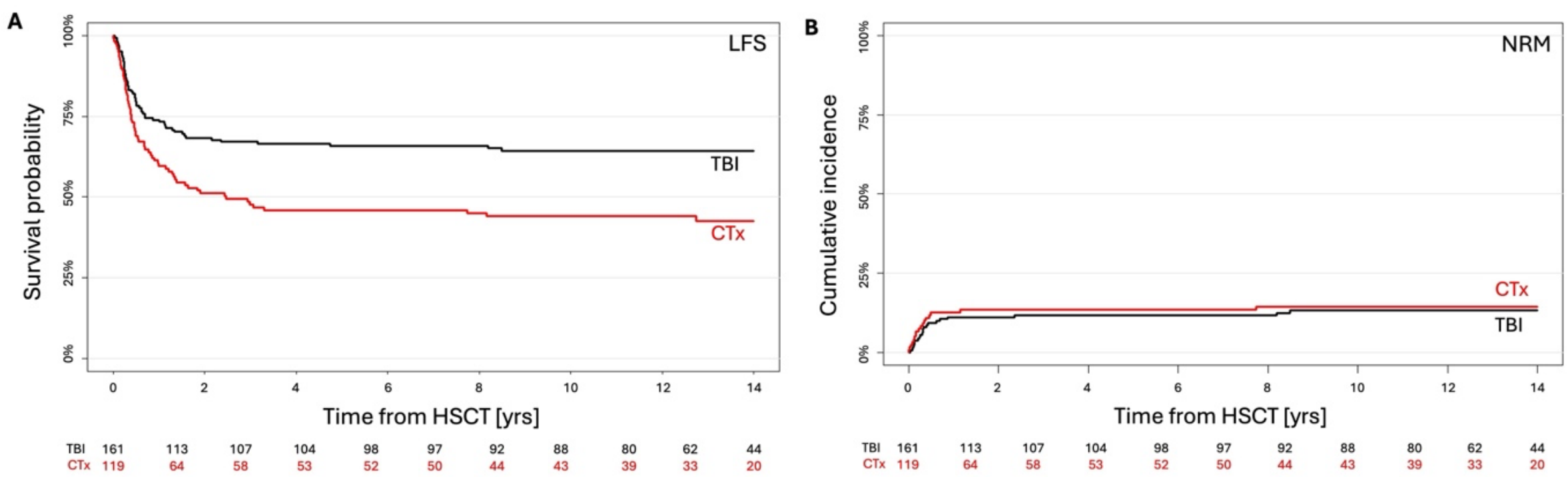

Supp. Figure 3. Cumulative incidence of GVHD. *Subgroups are indicated via color-code: CTx red, TBI black. A) aGVHD Grade II/IV, B) aGVHD Grade III/IV, C) cGVHD, D) extended cGVHD. Abbreviations: a/cGVHD acute/chronic graft-versus-host disease, CTx chemotherapy, HSCT hematopoietic stem cell transplantation, TBI total body irradiation, yrs years.*

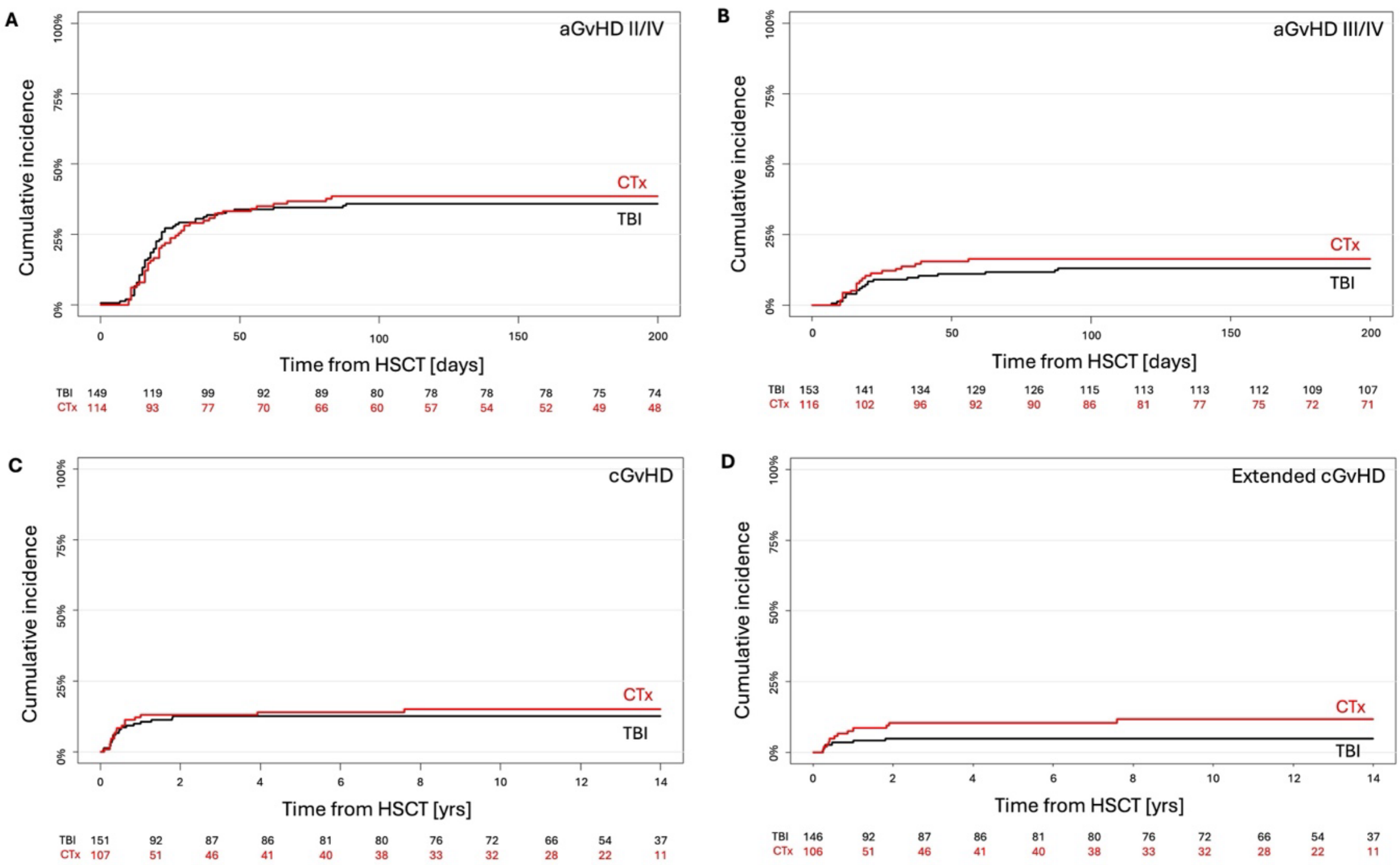

Supplement: Supplementary file 1 [file 41375_2026_3114_MOESM1_ESM.pdf]
